# Supplementary material for: Electrosprayed Agar Nanocapsules as Edible Carriers of Bioactive Compounds
Source: Foods. 2022 Jul 14;11(14):2093. doi: 10.3390/foods11142093 (PMC9319333; doi:10.3390/foods11142093)
Supplement: Supplementary file 1 [file foods-11-02093-s001.zip › foods-1751089-supplementary.pdf]

foods MDPI

Supplementary Materials

Electrosprayed Agar Nanocapsules as Edible Carriers of Bioactive Compounds

Barbara Tomadoni [1], María José Fabra [2,3], Daniel Alexander Méndez [2,4], Antonio Martínez-Abad [2,3] and Amparo López-Rubio [2,3,*]

[1] Grupo de Materiales Compuestos Termoplásticos (CoMP), Instituto de Investigaciones en Ciencia y Tecnología de Materiales (INTEMA), Universidad Nacional de Mar del Plata (UNMdP) y Consejo Nacional de Investigaciones Científicas y Técnicas (CONICET), Av. Colón 10850, Mar del Plata 7600, Argentina; btomadoni@gmail.com
[2] Packaging Group, Food Safety and Preservation Department, Institute of Agrochemistry and Food Technology (IATA-CSIC), Catedrático Agustín Escardino Benlloch 7, 46980 Paterna, Spain; mjfabra@iata.csic.es (M.J.F.); daamendezre@iata.csic.es (D.A.M.); conaba@iata.csic.es (A.M.-A.)
[3] Interdisciplinary Platform for Sustainable Plastics towards a Circular Economy-Spanish National Research Council (SusPlast-CSIC), 26006 Madrid, Spain
[4] Grupo de Investigación Bioecono, Facultad de Ciencias Económicas y Administrativas, Universidad del Tolima, 730006299, Tolima, Colombia
* Correspondence: amparo.lopez@iata.csic.es

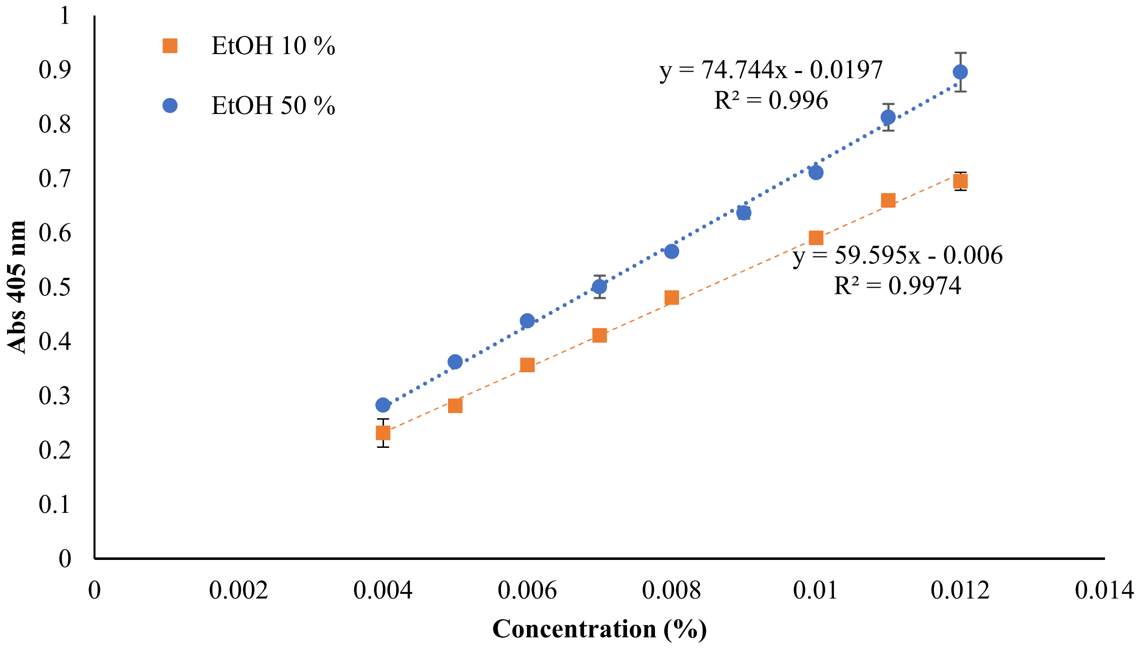

Figure S1. Chlorophyllin calibration curves in EtOH at 10 and 50% *v*/*v*.

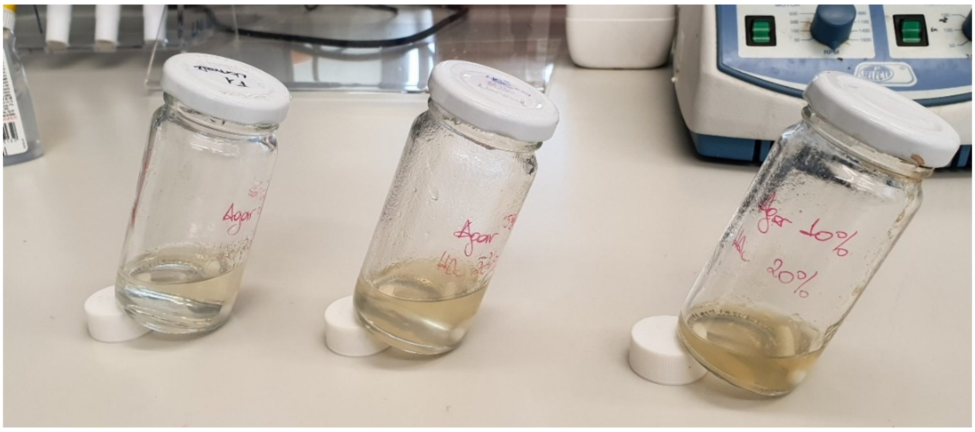

Figure S2. Agar solutions (from left to right: 2.5, 5.0 and 10.0% *w*/*v*) in HAc 20% *v*/*v*.

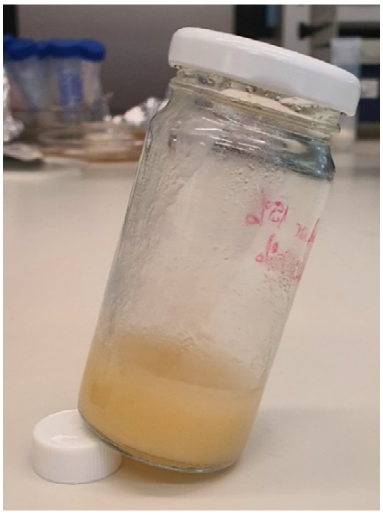

Figure S3. Agar 15 % *w*/*v* in HAc 20 % *v*/*v*.

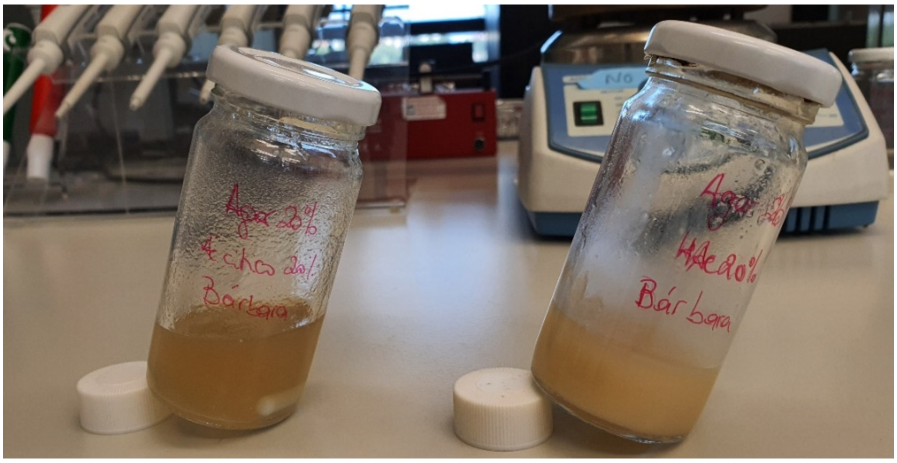

Figure S4. Evaluation of citric acid (left) and acetic acid (right) for the dissolution of agar 20% *w*/*v*.

Table S1. Mw weight average, Mn Number average and PDI polydispersity index, for agar samples prepared under different conditions.

Sample	Mn	Mw	PDI
Agar10%HAc20%	2.27	4.70	2.07
Agar15%HAc30%	2.29	5.52	2.27
Agar20%HAc40%	2.23	5.49	2.45
Agar15%HAc40%	2.26	5.56	2.45
Agar10%HAc40%	1.68	3.83	2.27
Agar10% - water	7.92	20.75	2.46

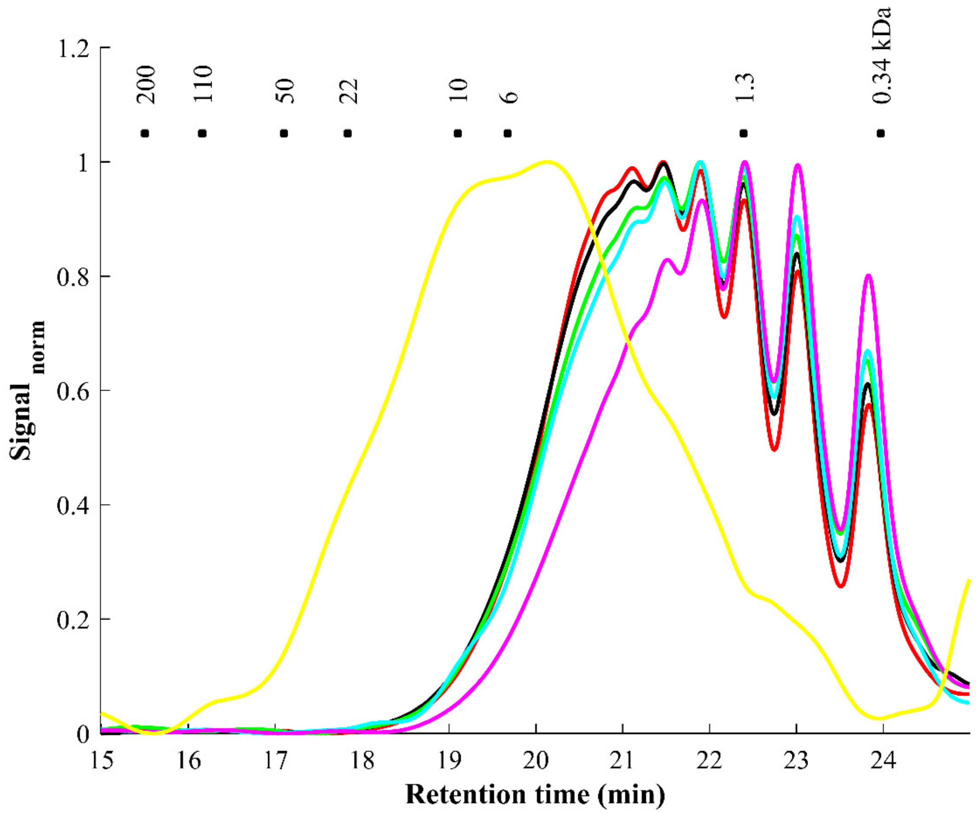

Figure S5. Molecular weight distribution of the agar solutions: Agar10%HAc20% ( ), Agar15%HAc30% ( ), Agar20%HAc40% ( ), Agar15%HAc40% ( ), Agar10%HAc40% ( ) and Agar10%-water ( ). The black squares correspond to pullulan standards with the matching molecular weight.
